# Naringenin Inhibits Adipogenesis and Reduces Insulin Sensitivity and Adiponectin Expression in Adipocytes

**DOI:** 10.1155/2013/549750

**Published:** 2013-07-29

**Authors:** Allison J. Richard, Zhaleh Amini-Vaughan, David M. Ribnicky, Jacqueline M. Stephens

**Affiliations:** ^1^Pennington Biomedical Research Center, Louisiana State University, 6400 Perkins Road, Baton Rouge, LA 70808, USA; ^2^Biotech Center, Rutgers University, New Brunswick, NJ 08901, USA

## Abstract

Adipose tissue development and function are widely studied to examine the relationship between obesity and the metabolic syndrome. It is well documented that the inability of adipose tissue to properly increase its lipid storage capacity during the obese state can lead to metabolic dysfunction. In a blind screen of 425 botanicals, we identified naringenin as an inhibitor of adipocyte differentiation. Naringenin is one of the most abundant citrus flavonoids, and recent studies have demonstrated antihyperlipidemic capabilities. These studies have largely focused on the effects of naringenin on the liver. Our biochemical studies clearly demonstrate that naringenin inhibits adipogenesis and impairs mature fat cell function. Naringenin specifically inhibited adipogenesis in a dose-dependent fashion as judged by examining lipid accumulation and induction of adipocyte marker protein expression. In mature 3T3-L1 adipocytes, naringenin reduced the ability of insulin to induce IRS-1 tyrosine phosphorylation and substantially inhibited insulin-stimulated glucose uptake in a dose-dependent manner and over a time frame of 1.5 to 24 hours. Exposure to naringenin also inhibited adiponectin protein expression in mature murine and human adipocytes. Our studies have revealed that naringenin may have a negative impact on adipocyte-related diseases by limiting differentiation of preadipocytes, by significantly inducing insulin resistance, and by decreasing adiponectin expression in mature fat cells.

## 1. Introduction

Adipocytes, once thought to be passive sites of lipid storage, are now recognized as dynamic insulin-sensitive cells with endocrine properties that contribute to whole-body energy homeostasis. Obesity, the primary disease of fat cells, is a major risk factor of metabolic syndrome and significantly contributes to the development of type 2 diabetes mellitus (T2DM), cardiovascular disease, and certain cancers (reviewed in [1, 2]). Limiting the development of adipose tissue was once considered a good way to combat obesity. In fact, many researchers investigated antiadipogenic agents as potential therapeutics for decreasing or preventing obesity. However, the prevailing current hypothesis is that disruption of adipocyte differentiation limits adipose tissue expansion, which is linked to insulin resistance and the development of T2DM [3–5]. 

In the United States and worldwide, awareness of the economic burden and health consequences associated with the growing obesity epidemic is rising [6]. This increased awareness, accompanied by dissatisfaction with conventional pharmaceuticals in dealing with chronic conditions, such as obesity and metabolic syndrome, has prompted interest among researchers and the general public in herbal or botanical remedies to treat these conditions [7]. However, often the safety and mechanism of action of the individual bioactive constituents are not well defined. 

Our laboratory participated in a blinded screening study to investigate the ability of over 400 botanicals to modulate adipocyte differentiation. Although less than 2% of the plant extracts or pure phytochemicals in our screen had substantial effects on adipogenesis, we identified naringenin to be antiadipogenic. 

Naringenin is an abundant aglycone flavanone found in grapefruit and other citrus fruits, and it has been recently reported to have anti-hyperlipidemic [8] and anti-hyperglycemic properties [9]. In contrast to these potential metabolically beneficial effects, our screening studies revealed that naringenin inhibited adipogenesis, indicating possible detrimental effects on adipose tissue expansion and insulin sensitivity. In these studies, we have evaluated the specific effects of naringenin on adipocyte development. Additionally, we tested the hypothesis that naringenin would also have negative effects on insulin sensitivity and adipokine production in mature mouse and human adipocytes. 

## 2. Materials and Methods

### 2.1. Materials

Dulbecco's modified Eagle's medium (DMEM) was purchased from Sigma-Aldrich (St. Louis, MO, USA). Bovine and fetal bovine sera were purchased from HyClone (Thermo Scientific, Logan, UT, USA). Naringenin was purchased from Sigma-Aldrich and also was provided as a compound purified from a botanical extract at the Rutgers Botanical Core facility. For immunoblotting, the polyclonal STAT5A and MAPK (ERK 1) IgG's and the PPAR*γ* monoclonal antibody were purchased from Santa Cruz Biotechnology. The polyclonal anti-aP2 (FABP4) and antiadiponectin antibodies were purchased, respectively, from Abcam (Cambridge, MA, USA) and Pierce (Thermo Scientific, Rockford, IL, USA). This polyclonal antiadiponectin antibody is reactive only against murine adiponectin. Therefore, to probe the whole cell extracts from human adipocytes, we used a monoclonal antiadiponectin IgG from Thermo Scientific that is reactive against both murine and human adiponectin. Antiphospho-IRS-1 (Tyr^896^), which is polyclonal and highly specific for phosphotyrosine 896 of the mouse IRS-1, was purchased from Invitrogen (Carlsbad, CA, USA). The BCA and enhanced chemiluminescence kits were from Pierce. Anti-rabbit and anti-mouse horseradish peroxidase-conjugated secondary antibodies were purchased from Jackson ImmunoResearch Laboratories. Mature human subcutaneous adipocytes were purchased in a 12-well plate format from Zen-Bio. These mature human adipocytes were derived from preadipocytes collected from the subcutaneous thigh region of nondiabetic females with a BMI of less than 25. 

### 2.2. Cell Culture

Murine 3T3-L1 preadipocytes were plated and grown to 2 days after confluence in DMEM containing 10% bovine serum. Medium was changed every 48–72 hours. Cells were induced to differentiate by changing the medium to DMEM containing a standard MDI induction cocktail of 10% fetal bovine serum (FBS), 0.5 mM 3-isobutyl-1-methylxanthine, 1 *μ*M dexamethasone, and 1.7 *μ*M insulin. After 48 h, this medium was replaced with DMEM supplemented with 10% FBS and 0.43 *μ*M insulin. Each additional feeding occurred every 2-3 days in DMEM supplemented with 10% FBS, and cells were maintained in this medium until utilized for experimentation. Human subcutaneous adipocytes were maintained in adipocyte maintenance media (Zen-BIO Cat # AM-1) and used for experimentation within one week of arrival.

### 2.3. Whole Cell Extract Preparation

Monolayers of 3T3-L1 adipocyte cells were harvested in a nondenaturing buffer containing 150 mM NaCl, 10 mM Tris, pH 7.4, 1 mM EGTA, 1 mM EDTA, 1% Triton X-100, 0.5% IGEPAL CA-630, 1 *μ*M phenylmethylsulfonyl fluoride, 1 *μ*M pepstatin, 1 mM 1,10-phenanthroline, 50 trypsin inhibitory milliunits of aprotinin, 10 *μ*M leupeptin, and 2 mM sodium vanadate. The samples were either extracted on ice for 30 min or frozen at −80°C. Following the incubation period or thawing of frozen samples, the samples were centrifuged at 13 000 ×g at 4°C for 10 min. Supernatants containing whole cell extracts were analyzed for protein content using a BCA kit according to the manufacturer's instructions. 

### 2.4. Gel Electrophoresis and Immunoblotting

Proteins were separated on 7.5%, 10%, or 15% polyacrylamide (acrylamide from National Diagnostics) gels containing SDS according to Laemmli [10] and transferred to a nitrocellulose membrane in 25 mM Tris, 192 mM glycine, and 20% methanol. Following transfer, the membrane was blocked in 4% milk overnight at 4°C or for 1.5 h at room temperature and then immunoblotted. Results were visualized with horseradish peroxidase-conjugated secondary antibodies and enhanced chemiluminescence. Densitometric analysis of western blot band intensities was performed using ImageJ [11]. 

### 2.5. Oil Red O Staining

An Oil Red O stock was prepared as previously described [12]. Cell monolayers were aspirated and rinsed with PBS. Following incubation in a fixative solution (10% formaldehyde in PBS) for 10–15 min, the monolayers were rinsed 5 times under tap water. The remaining water was aspirated, and the cells were incubated for 1 h in the working Oil Red O solution (0.3% in isopropanol). Following incubation, the stain was aspirated, the cells were rinsed 5 times under tap water, and then they were examined by eye and microscopy. For all treatment conditions, the monolayer was monitored by microscopy and not substantially disturbed as a result of the staining and aspiration procedure. 

### 2.6. Insulin Stimulated 2-[^3^H] Deoxyglucose Uptake Assay

The assay of 2-[^3^H] deoxyglucose uptake was performed as previously described [13]. Prior to the assay, mature 3T3-L1 adipocytes were serum-deprived for 24 h and pretreated with naringenin for various times. Next, the cells were incubated in the presence or absence of insulin (5 nM) for 7 min. Glucose uptake was initiated by addition of 2-[^3^H] deoxyglucose at a concentration of 0.1 mM 2-deoxyglucose in 1 mCi 2-[^3^H] deoxyglucose in Krebs-Ringer-Hepes buffer and incubated for 3 min at room temperature. Glucose uptake is reported as [^3^H] radioactivity, normalized to total protein content as determined by BCA analysis. Nonspecific uptake and absorption were always less than 10% of the total uptake. Uptake measurements were performed in triplicate under conditions where hexose uptake was linear. 

### 2.7. Statistical Methods

All statistical analyses were performed using the statistical software SAS 9.3 (SAS Institute, Inc., Cary, NC, USA). Each experiment was conducted as a completely randomized design using a factorial arrangement of interventions, and separate analyses were performed accordingly. The general linear models procedure was employed to conduct an analysis of variance for glucose uptake with insulin at (i) 4 concentrations of naringenin and (ii) 4 treatment lengths with a control for each level of factors (i) and (ii). Results were summarized as least squares means and standard deviations. As expected, the differences in glucose uptake with and without insulin varied significantly across both factors, necessitating *post hoc* pairwise comparisons to characterize the specific findings. The resulting *P* values were conservatively adjusted using the method of Scheffé to control the global significance level at 0.05 for each experiment. Thus, statistical significance was declared for comparisons with adjusted *P* values ≤ 0.05.

## 3. Results

### 3.1. Naringenin Inhibits Adipogenesis of 3T3-L1 Cells

**Figure 1:**
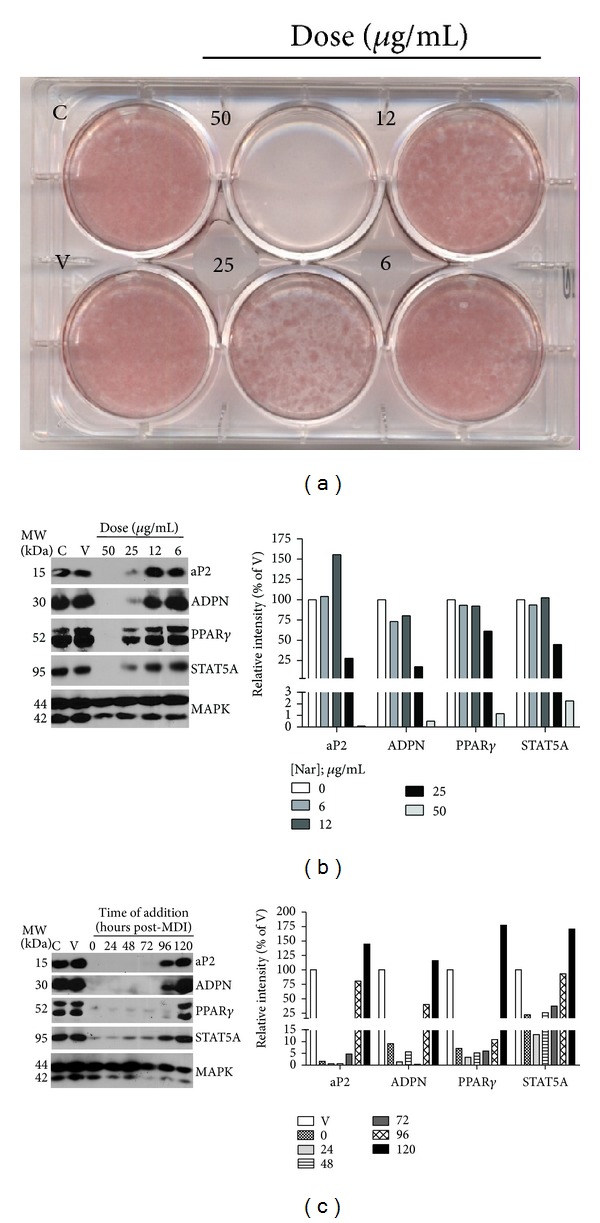
Naringenin blocks differentiation of murine 3T3-L1 preadipocytes. Preadipocytes were induced to differentiate using the typical MDI induction cocktail. Immediately following induction ((a) and (b)) or at the indicated time point post-MDI (c), cells were treated with DMSO vehicle (V), the indicated the dose of naringenin ((a) and (b)), or 25 *μ*g/mL naringenin (c). The cells were retreated with the indicated dose of naringenin following each media change every 48–72 hours as indicated in the methods. The control cells (C) received no treatment. Each panel is representative of 3–6 independently performed experiments. Oil Red O staining of neutral lipid content was performed 7 days post-MDI (a). Fifty to one hundred fifty micrograms of protein from whole cell extracts, harvested at four (b) or seven (c) days post-MDI were subjected to western blot analysis. Band intensities were quantified, normalized against the loading control, MAPK, and presented as a bar graph next to each immunoblot. Similar dose-dependent patterns of adipocyte marker protein expression were observed when cells were harvested at either 4 or 7 days post-MDI, and panel (b) contains a representative subset of these blots.

We performed a blinded screen of 425 botanical extracts or phytochemicals for their ability to modulate adipocyte differentiation of 3T3-L1 cells. We observed that less than 2% of the extracts had substantial effects on adipocyte differentiation. One of the botanicals we screened that inhibited adipogenesis was naringenin. As shown in Figure 1(a), naringenin resulted in a dose-dependent inhibition of adipogenesis of 3T3-L1 cells as judged by lipid accumulation. There was no detectable difference in Oil Red O (ORO) staining between control (C) and DMSO vehicle- (V-) treated cells. Following ORO staining, we assessed each well using phase contrast microscopy and ensured that the integrity of the monolayer was not substantially disturbed. When 3T3-L1 preadipocytes were exposed to the differentiation cocktail and 50 *μ*g/mL naringenin, the cells maintained a fibroblastic appearance (data not shown) and did not accumulate lipid (Figure 1(a)). Although the 25 *μ*g/mL dose resulted in a substantial inhibition of lipid accumulation, there was no notable effect on Oil Red O staining at the lowest naringenin dose (6 *μ*g/mL) compared to control and vehicle-treated cells. When the cells were exposed to naringenin, we did not observe cytotoxic effects as monitored by microscopic visualization of monolayer integrity (data not shown). This is in agreement with a previous study [14] that used lactate dehydrogenase release to monitor the effect of naringenin and other flavonoids on the viability of postconfluent, differentiating 3T3-L1 cells.

Adipogenesis was also assessed by examining the induction of adipocyte marker proteins. As shown in Figure 1(b), the higher doses of naringenin blocked the induction of aP2, PPAR*γ*, STAT5A, and adiponectin expression that normally accompanies adipogenesis. The lower doses of naringenin did not inhibit the induction of these adipocyte markers. Vehicle treatment of DMSO had no effect on the expression of adipogenic marker proteins. MAPK expression was measured to ensure equivalent levels of protein in each sample.

Next, we examined the ability of naringenin to inhibit differentiation when added at various times after the induction of adipogenesis. As indicated in Figure 1(c), 3T3-L1 preadipocytes were induced to differentiate and naringenin (25 *μ*g/mL) was added to the induction cocktail at the time of induction (time 0) and at different time points after induction of differentiation (24, 48, 72, 96, or 120 hours). At 168 hours (7 days), whole cell extracts were isolated from the cells in which naringenin treatment was initiated at the six different time points, as well as from untreated controls and vehicle-treated cells. As shown in Figure 1(c), naringenin effectively blocked adipogenesis for all treatment periods that began up to 72 h post-MDI. The effects of naringenin on the expression of adipocyte marker proteins, with the exception of PPAR*γ*, were substantially attenuated when treatment began at 96 h post-MDI, and there were no observable differences in adipocyte marker protein expression at 120 h (Figure 1(c)). 

### 3.2. Naringenin Decreases Insulin Sensitivity of Mature Adipocytes

**Figure 2:**
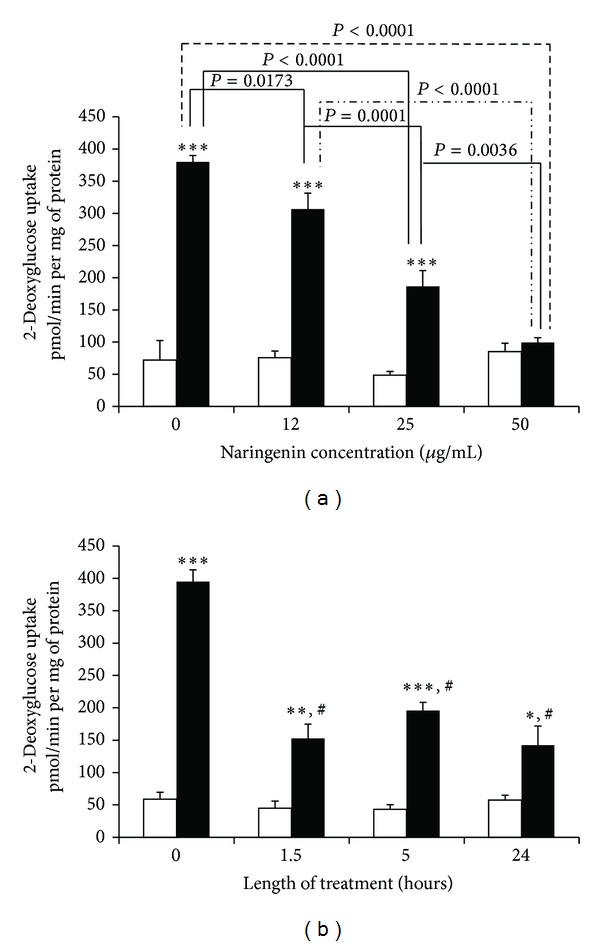
Naringenin inhibits insulin-sensitive glucose uptake from 1.5 to 24 hours and in a dose-dependent manner. Fully differentiated 3T3-L1 adipocytes were serum-deprived for 24 hours prior to performing glucose uptake assays. Cells were stimulated with 5 nM insulin for 7 minutes. Glucose uptake was initiated by the addition of 2-[^3^H] deoxyglucose. The glucose uptake values shown represent the mean ± SEM of triplicate determinations from three independent experiments. Cells were pretreated with vehicle (DMSO) or the indicated dose of naringenin overnight (a). Prior to insulin administration, the serum-deprivation medium was refreshed and the cells were retreated with the vehicle and indicated dose of naringenin. Cells were treated with vehicle (0 time point) for 24 hours or 50 *μ*g/mL of naringenin for the indicated length of time prior to insulin administration (b). White bars: minus insulin; black bars: plus insulin. Glucose uptake was significantly greater with insulin (relative to basal uptake): *P = 0.002, **P = 0.0002, and ***P < 0.0001. Statistically significant relationships among insulin-stimulated glucose uptake values are indicated in (a) for all combinations of naringenin doses. ^#^Represents statistical significance (*P* < 0.0001) for each naringenin versus 0 time point. All other comparisons were not statistically significant.

**Figure 3:**
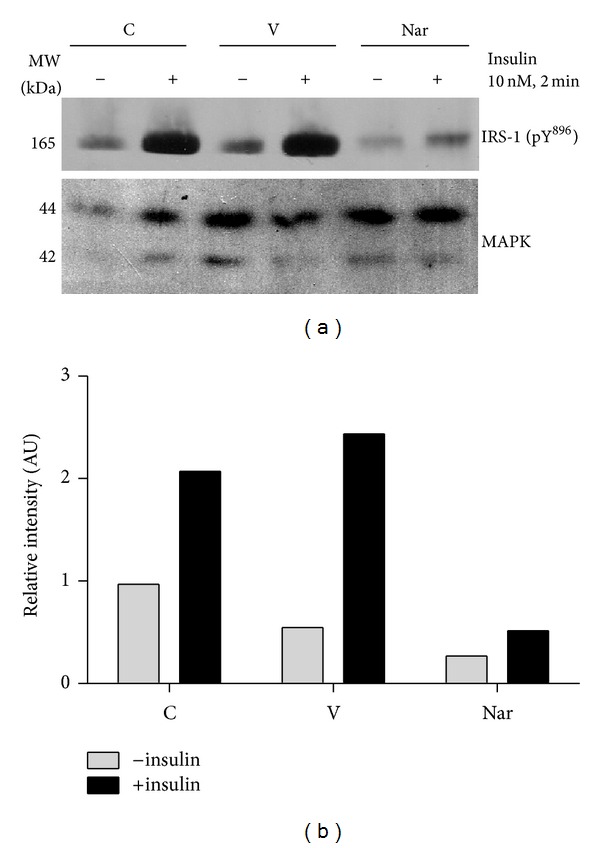
Naringenin modulates insulin action via attenuation of IRS-1 tyrosine^896^ phosphorylation. Mature 3T3-L1 adipocytes were serum deprived overnight and then pretreated with vehicle or 25 *μ*g/mL naringenin (Nar) for 2 hours. The control (C) cells were untreated. As indicated, cells were then stimulated with 10 nM insulin for 2 minutes prior to preparation of whole cell extracts. One hundred fifty micrograms of protein from the whole cell extracts were separated by SDS-PAGE, transferred to nitrocellulose, and probed for IRS-1 tyrosine^896^ phosphorylation using a phosphospecific antibody directed against this site (a). Band intensities were quantified, normalized against the loading control, MAPK, and presented as a bar graph (b). This experiment was independently repeated three times.

**Figure 4:**
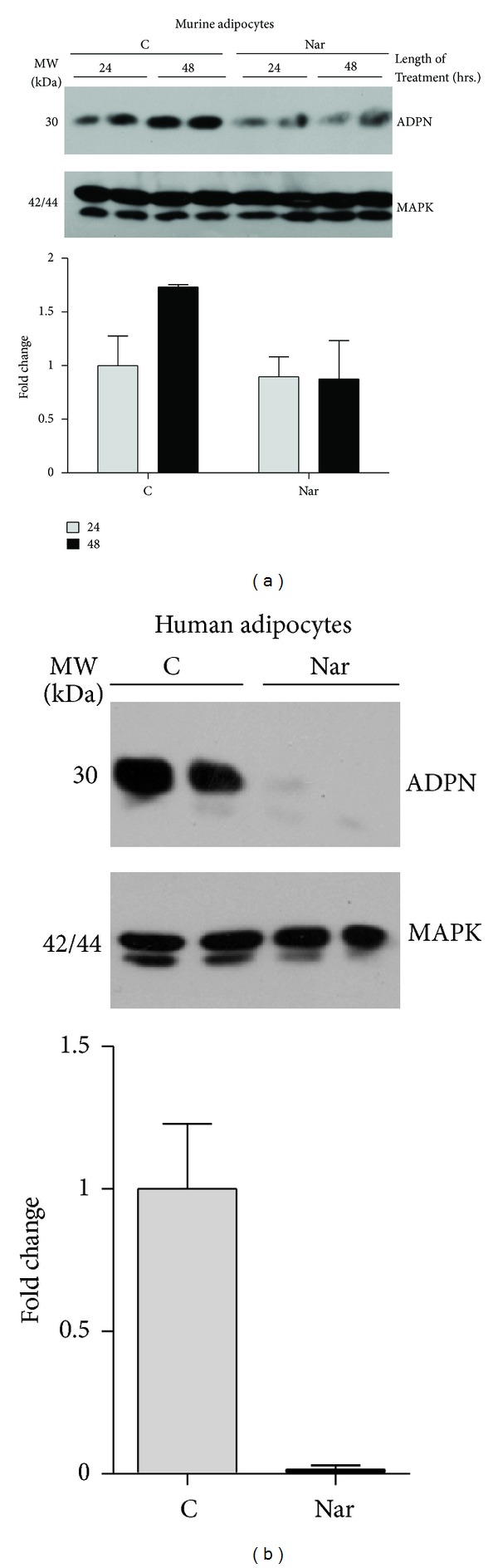
Naringenin inhibits adiponectin expression in mature 3T3-L1 and subcutaneous human adipocytes. Mature 3T3-L1 adipocytes cultured in 6 well plates were treated with 50 *μ*g/mL naringenin (Nar) or DMSO vehicle control (C) for the indicated length of time prior to the preparation of whole cell extracts (a). For each time point, data from two individual wells are shown. Subcutaneous human adipocytes cultured in 12 well plates were treated with 25 *μ*g/mL naringenin (Nar) for 72 hours prior to harvesting (b). The control cells (C) received no treatment. For the 48- and 72-hour treatments, cells were treated daily, and the medium was replaced 24 hours prior to harvesting the whole cell extracts. Fifty to one hundred fifty micrograms of protein from whole cell extracts were separated by SDS-PAGE, transferred to nitrocellulose, and used for western blot analysis. The anti-mouse adiponectin antibody was used to probe the samples in (a), whereas the anti-mouse/human adiponectin antibody was use to probe the samples in (b). Band intensities were quantified, normalized against the loading control, MAPK, and presented as fold change relative to 24 h control (a) or control (b) in a bar graph beneath each immunoblot. Values for duplicate samples were averaged, and data are presented as mean ± SEM.

Since naringenin negatively affected adipocyte development, we hypothesized that it may also impair the physiology of fully differentiated adipocytes. To assess mature adipocyte function, we investigated insulin sensitivity (Figures 2 and 3) and adiponectin production (Figure 4). For the insulin sensitivity studies, we preincubated mature 3T3-L1 adipocytes with naringenin or vehicle (indicated as 0 in Figures 2(a) and 2(b)) and then monitored the uptake of [H^3^]-labeled 2-deoxyglucose in the presence and absence of an acute insulin dose. As shown in Figure 2, vehicle-treated adipocytes were highly responsive to insulin treatment, which induced a 5-6-fold increase in glucose uptake compared to the basal rate. Although basal rates of glucose uptake were not significantly altered, overnight preincubation with naringenin specifically attenuated insulin-stimulated glucose uptake in a dose-dependent manner (Figure 2(a)). At the highest concentration tested, naringenin completely blocked the insulin-induced uptake of glucose into the fat cells, and at the lowest concentration, it exhibited a minimal but statistically significant inhibitory effect. We also tested the ability of naringenin to alter glucose uptake by treating the adipocytes with 50 *μ*g/mL naringenin for various lengths of time. As shown in Figure 2(b), naringenin was capable of considerably inhibiting insulin-stimulated glucose uptake in mature 3T3-L1 adipocytes with a treatment period as short as 1.5 hours. This inhibitory effect was maintained at 5 and 24 hours. A similar inhibitory response was observed when the cells were treated with a 25 *μ*g/mL dose of naringenin over the same time frame—1.5 to 24 hours (data not shown).

To investigate how naringenin disrupts insulin signaling in fat cells, we examined its ability to inhibit tyrosine phosphorylation of IRS-1. IRS-1 is a primary substrate of the insulin receptor. Tyrosine phosphorylation of IRS-1 is crucial in its ability to transmit insulin's signal and to activate downstream insulin-responsive pathways, such as the PI3 K/Akt signaling pathway [15]. Using a phosphospecific anti-IRS-1 pY^896^ antibody, we monitored the effect of naringenin on IRS-1 pY^896^ in mature 3T3-L1 adipocytes following acute administration of insulin. As shown in Figure 3, we observed a robust increase in phosphorylation of IRS-1 at Tyr^896^ following insulin stimulation of untreated control cells. Although the insulin response of the vehicle-treated cells was similar to that of the untreated cells, a 2 h naringenin pretreatment resulted in a significantly lower level of insulin-induced IRS-1 tyrosine phosphorylation. In fact, the amount of activated IRS-1 in the insulin-stimulated, naringenin-treated cells was comparable to that of the basal levels observed in the control and vehicle-treated fat cells. In Figure 3, naringenin had a modest effect on the amount of un-stimulated, basal IRS-1 tyrosine phosphorylation relative to control and vehicle treatments. However, this decrease was not consistently observed, and nonetheless, naringenin reduced the relative phosphoactivation IRS-1 (insulin-stimulated versus basal) from 4.4-fold in vehicle-treated cells to 1.9-fold. MAPK expression was not altered by acute insulin stimulation and was examined as a loading control for this experiment. Similar to its effect on insulin-stimulated glucose uptake, naringenin also acutely inhibited IRS-1 phosphorylation at 1 hour, and this response was maintained over a 24-hour period (data not shown). We consistently observed that human adipocytes were generally less insulin-sensitive than the 3T3-L1 fat cells. Nonetheless, we found that naringenin also attenuated Akt Ser^473^ phosphorylation in human adipocytes (data not shown).

### 3.3. Naringenin Attenuates Adiponectin Expression in 3T3-L1 and Human Adipocytes

Decreased expression and/or secretion of adiponectin, an adipocyte-specific hormone, strongly correlates with whole-body insulin resistance [16]. Thus, we examined the ability of naringenin to modulate adiponectin expression in mature 3T3-L1 adipocytes. As shown in Figure 4(a), 3T3-L1 adipocytes treated with vehicle for 48 hours exhibited slightly higher adiponectin expression than those treated for 24 hours. At the 24-hour time point, naringenin did not affect adiponectin expression relative to vehicle-treated fat cells. However, at 48 hours, naringenin substantially inhibited the time-dependent increase in adiponectin protein expression observed under vehicle conditions. Duplicate samples at each time point for the vehicle and naringenin treated cells are shown. We also monitored the effect of naringenin on adiponectin expression in subcutaneous human adipocytes. Human adipocytes were untreated or treated with 25 *μ*g/mL naringenin for 72 hours, and then the whole cell extracts were analyzed for adiponectin content. As shown in Figure 4(b), naringenin treatment robustly reduced adiponectin protein levels in human fat cells. These experiments were performed in duplicate and yielded similar results. For Figures 4(a) and 4(b), MAPK expression was examined to demonstrate equivalent protein levels in each sample. 

## 4. Discussion

In a blinded screen to identify novel modulators of adipocyte differentiation, we observed that naringenin, an abundant grapefruit flavanone, inhibited adipogenesis. Using 3T3-L1 cells as a model system, we demonstrated that naringenin specifically suppressed lipid accumulation and the induction of adipocyte-specific marker proteins in a dose-dependent manner. Subsequent to the administration of the MDI cocktail, growth-arrested postconfluent 3T3-L1 preadipocytes undergo mitotic clonal expansion (MCE) prior to the exiting cell cycle and expressing genes that yield the mature adipocyte phenotype [17–19]. Mitotic clonal expansion plays an essential role in adipogenesis of 3T3-L1 cells and occurs within 48–72 hours after MDI stimulation [19]. Many reagents that inhibit adipogenesis do so by inhibiting this proliferative phase. Interestingly, in a previous study on the ability of flavonoids to modulate proliferation and adipogenesis of 3T3-L1 cells, it was shown that naringenin exerted substantial antiproliferative effects during the pre-confluent phase, but was unable to modulate MCE and differentiation of 3T3-L1 preadipocytes [14]. 

We did not examine the effects of naringenin on proliferating preadipocytes or proliferation during the early stages of adipogenesis. However, we did monitor the ability of naringenin to inhibit fat cell development when added at different time points following the induction of adipogenesis. Naringenin potently blocked differentiation when administration began any time within the clonal proliferative phase, the first 3 days following MDI stimulation. Furthermore, when treatment of the cells began at 4 days post-MDI, naringenin was able to significantly attenuate adipogenesis, indicating that it is capable of exerting its inhibitory action at later stages of the differentiation process occurring after clonal expansion. 

In addition to its ability to block adipogenesis of 3T3-L1 preadipocytes, our studies demonstrated that naringenin also substantially inhibited insulin-sensitive glucose uptake in fully differentiated adipocytes. This effect was dose dependent and consistent when fat cells were exposed to naringenin from 1.5 to 24 hours. Pretreatment of murine fat cells with 50 *μ*g/mL naringenin for 1.5, 5, and 24 hours considerably reduced insulin-sensitive glucose uptake. Although 12 *μ*g/mL naringenin resulted in minimal inhibition of adipogenesis, this dose was capable of significantly attenuating glucose uptake induced by acute insulin treatment, suggesting that mature adipocytes are more sensitive than preadipocytes to the effects of naringenin. Several studies in 3T3-L1 [20, 21] and human adipocytes [20] support our finding that naringenin impairs insulin-sensitive glucose uptake. Our data are novel because they indicate that the ability of naringenin to attenuate insulin sensitivity in mature fat cells is more potent than its ability to impair adipogenesis or lipid accumulation in differentiating adipocytes. 

In adipocytes and skeletal muscle, insulin-stimulated glucose uptake is mediated via translocation of GLUT4 to the plasma membrane from intracellular storage vesicles. Naringenin inhibits insulin-responsive glucose transport in 3T3-L1 adipocytes both directly via interaction with the GLUT4 transporter and indirectly via impairment of insulin signaling processes involved in GLUT4 trafficking [20]. Disruption of the insulin signaling pathway by naringenin is also supported by data demonstrating that naringenin can inhibit PI3 K activity and phosphorylation of its downstream target, Akt [21]. Activation of PI3 K and Akt is crucial for insulin-responsive glucose uptake [22–27]. 

To further investigate the effects of naringenin on the insulin signaling cascade in fat cells, we examined its ability to modulate IRS-1 phosphorylation at tyrosine^896^. IRS-1 is a key protein upstream of PI3 K and Akt, and inhibition of its expression or tyrosine phosphorylation is often associated with insulin resistance [15]. Our studies demonstrated that naringenin significantly reduced insulin-induced phosphorylation of IRS-1 at tyrosine^896^ in 3T3-L1 adipocytes. Of note, a previous investigation reported that the inhibitory effect of naringenin on insulin signaling in adipocytes was not mediated by modulation of insulin-sensitive IRS-1 tyrosine phosphorylation [21]. The observed disparity in the ability of naringenin to modulate IRS-1 tyrosine phosphorylation between our study and that of Harmon and Patel is likely attributed to differences in the specificities of the antibodies used to detect phosphorylated IRS-1, which contains over 20 potential tyrosine phosphorylation sites [28, 29]. 

Overall, our results support the hypothesis that naringenin inhibits insulin-sensitive glucose uptake at least partially via its ability to impair insulin signaling. In addition to its ability to attenuate Akt Ser^473^ phosphorylation, our data demonstrate that naringenin also can act upstream of Akt and inhibit insulin-sensitive IRS-1 Tyr^896^ phosphorylation in 3T3-L1 adipocytes. Although skeletal muscle is proposed to be primarily responsible for glucose disposal following a meal [30], studies have shown that adipose-selective disruption of the GLUT4 gene weakens the insulin response in the other insulin-sensitive tissues (muscle and liver), suggesting that adipose tissue plays an important role in systemic glucose homeostasis [31]. Thus, we propose that naringenin may be capable of having a negative impact on whole-body glucose homeostasis via its ability to induce insulin resistance in adipocytes. Indeed, a recent study showed that orally administered naringenin impaired systemic glucose tolerance in mice and hamsters possibly via inhibition of hypothalamic insulin signaling [32]; however, insulin-responsiveness in adipose tissue was not examined. 

A previous study measured the plasma concentration of naringenin in human subjects following consumption of grapefruit juice to be 6*μ*M (1 *μ*g/mL) [33]. Based on the results of our study, naringenin did not inhibit adipogenesis at this concentration (Figure 1(a)), and the ability of naringenin to block insulin-stimulated glucose uptake in a dose-dependent manner suggests that naringenin would not substantially attenuate adipocyte insulin sensitivity following grapefruit juice consumption. However, several recent studies have proposed that naringenin might be therapeutically beneficial for the treatment of metabolic dysfunction associated with hyperlipidemia and hyperglycemia [8, 9, 34, 35]. Pharmacological levels of naringenin could be much higher than the levels attained following dietary consumption of naringenin-containing foods. Our data indicate that mature adipocytes are more sensitive to naringenin than differentiating 3T3-L1 cells and warrant further investigation of the effects of pharmacological doses of naringenin on adipose tissue *in vivo*. 

One method by which adipocytes can modulate systemic insulin sensitivity is via their ability to secrete hormones, like adiponectin. Decreased expression and/or secretion of adiponectin strongly correlates with whole-body insulin resistance [16]. We observed that naringenin decreased the protein expression of adiponectin in mouse and human adipocytes, suggesting that it may modulate insulin action in other tissues. Notably, in contrast to our observations, other studies indicated that naringenin increases adiponectin gene expression [36, 37] and protein secretion [36]. Although our results do not correlate with the data from these studies, it is not uncommon for gene and protein expression levels to differ. Additionally, we do not think that these inconsistencies in adiponectin expression are due to variation in the source of naringenin. In our experiments, we used naringenin from two separate sources, the Rutgers Botanical Center and Sigma-Aldrich. All of the experiments shown in this paper were performed with naringenin that was purchased from Sigma-Aldrich, but similar experiments with the Rutgers naringenin that was purified from a botanical extract yielded identical results (data not shown). 

Several studies have shown that naringenin has antidiabetic, anti-hyperlipidemic, and insulin-sensitizing effects *in vivo* using rodent models of metabolic dysfunction [8, 9, 34, 35]. These positive *in vivo* observations potentially contrast with the results of our current study and other previously reported* in vitro* observations [20, 21]. We hypothesize two possible theories to account for these divergences. First, it is possible that a metabolite of naringenin might be responsible for the *in vivo* improvement in metabolic parameters. Pharmacokinetic studies of naringenin in rats and humans have yielded controversial results. Although one study demonstrated high bioavailability of this flavanone aglycone in rats [38], other investigations indicated that its glucuronidated metabolite is well absorbed, but the bioavailability of naringenin is low [39, 40]. 

Alternatively, naringenin may induce insulin-sensitizing activity in muscle and liver or anti-inflammatory activity in adipose tissue that outweighs its potentially negative effects in isolated adipocytes. These hypotheses are supported by studies examining the effects of naringenin in myocytes and hepatocytes *in vitro *and adipose tissue* in vivo*. Naringenin has been shown to increase glucose uptake via an AMPK-dependent mechanism [41] and exhibit insulin-like effects in liver cells via a novel mechanism that results in PI3 K activation [23]. Moreover, dietary supplementation with naringenin reduced adipose tissue inflammation in a mouse model of dyslipidemia and metabolic syndrome [34]. Clearly, future *in vitro* and *in vivo* studies of naringenin and its metabolites are needed to better understand its mechanism(s) of action in insulin-sensitive cells and how its specific actions in various tissues might contribute to systemic effects *in vivo*. 

## 5. Concluding Remarks

Taken together, novel findings from our study demonstrate that naringenin is a potent botanically derived antiadipogenic factor that also negatively impacts adiponectin protein expression in mature adipocytes. While the effects of naringenin on adiponectin gene expression and protein secretion have been previously studied [36, 37], to our knowledge, our study is the first to show that naringenin inhibits adiponectin protein expression in fully differentiated adipocytes from both mouse and human. Grapefruit consumption is associated with many beneficial health effects [42–45]. Although naringenin is a major component of grapefruit and other citrus fruits, we are not proposing that grapefruit might be metabolically harmful. However, our studies do suggest that isolated naringenin may have a negative impact on adipocyte-related diseases by limiting differentiation of preadipocytes and by inducing insulin resistance and reducing adiponectin expression in mature fat cells.
